# Condom-Related Stigma Scale among Men Who Have Sex with Men in China: Development and Psychometric Tests

**DOI:** 10.3390/ijerph20064779

**Published:** 2023-03-08

**Authors:** Yan Shen, Ci Zhang, Lloyd A. Goldsamt, Wenwen Peng, Run Wang, Xianhong Li

**Affiliations:** 1 Xiangya School of Nursing, Central South University, Changsha 410013, China; yan_shen@csu.edu.cn (Y.S.);; 2 Rory Meyers College of Nursing, New York University, New York, NY 10010, USA

**Keywords:** scale development, psychometric test, condom-related stigma, HIV/AIDS, men who have sex with men

## Abstract

Condom-related stigma is a frequently mentioned barrier to consistent condom use among men who have sex with men (MSM). Based on the concept and operational definition of condom-related stigma recently defined by our team, we developed the 20-item condom-related stigma scale (CRSS) and examined its psychometric properties among 433 MSM in China, following DeVellis’s scale development guidelines. The content validity, convergent validity, empirical validity, factorial validity, scale score reliability, split-half reliability, and test–retest reliability for the CRSS were all assessed. The scale consists of four domains: perceived distrust, perceived potential HIV/STI risk, perceived embarrassment, and perceived violation of the traditional understanding of sexual intercourse. The CRSS has good validity (the scale-level content validity index was 0.99; the empirical validity was greater than 0.70) and high reliability (the Cronbach’s alpha coefficient overall was 0.926; the split-half reliability overall was 0.795; the test–retest reliability overall was 0.950). This scale is recommended for assessing the level of condom-related stigma among Chinese MSM, which can serve as an evaluating indicator for safer-sex interventions to prevent HIV infection among the MSM population in a Chinese cultural context.

## 1. Introduction

According to the Joint United Nations Programme on HIV/AIDS (UNAIDS), there were approximately 1.7 million people newly infected with HIV/AIDS in 2019 globally, and men who have sex with men (MSM) accounted for 23% of total new infections [1]. China also had a similar trend of HIV infection among MSM, with the rate of HIV infection increasing dramatically from 1.77% in 2000 to 6.9% in 2018 [2], and nearly one-third of new HIV infections attributed to MSM during recent years [3]. Evidence indicated that MSM were 26 times more likely to test positive for HIV compared to other adult men [1], due to a high frequency of unprotected sex, multiple sexual partners, and substance use [4,5].

Consistent condom use has been confirmed to effectively reduce the HIV risk among key populations, including MSM [6]. During the past 40 years, many intervention strategies were developed to increase consistent condom use among MSM, such as peer education [7], mass media education [8], condom social marketing [9], motivational interviewing [10], and psychosocial support and counseling services [9,11,12,13,14]. A systematic review showed HIV-prevention interventions increased willingness and awareness of condom use among MSM worldwide [15] and reduced occasions of unprotected anal sex by 27% (95% confidence interval [CI] = 15% to 37%) [15]. However, the effectiveness of these strategies in the real world is mixed; for instance, some systematic reviews and large-scale surveys show that the rate of consistent condom use is only 47% in China [16], 66% in the United States [17], 63% in Japan [18], 53.3% in Thailand [19], 65% in Kenya [20], and 51.6% in Cambodia [21], still far below the ultimate goal of 100% consistent condom use [18]. In recent years, pre-exposure prophylaxis (PrEP) was identified as an effective biological intervention to reduce the risk of HIV infection for those who were reluctant to use condoms [22]. The willingness to use PrEP by MSM was 58.6% worldwide [23] and 65.8%~84.9% in China [24,25,26]; however, the accessibility of PrEP remained low. Only 10% of people globally who were at risk could benefit from PrEP [27], and this proportion in China was even lower (4.3%) [28], due to the low awareness of PrEP, doubts about its efficacy and side effects, unaffordability, and HIV/AIDS stigma [24,27,29,30]. Therefore, promoting condom use during sex is still the dominant strategy to prevent HIV and other sexually transmitted infections (STIs) in the world, especially in low- and middle-income countries [31].

Many factors for inconsistently using condoms have been identified in the literature, including a lack of HIV/STI-related knowledge [32,33]; condoms being expensive [34,35]; physical discomfort [35,36]; and psychological factors [37,38,39]. Previous studies also indicated that initiating the discussion about using condoms was a barrier, mainly due to condom-related stigma. For example, using a condom might be considered as disloyal or indicate infection with HIV or a sexually transmitted infection [38,39,40,41], which would be presumed to be caused by MSM’s sociocultural and subcultural context [42,43]. Our team has recently comprehensively defined the concept of condom-related stigma by using concept analysis and meta-synthesis [44,45]. According to Goffman, stigma indicates an inherently undesirable characteristic or property in a particular population, which makes a person feel discreditable or shamed [46] (pp. 1–6). The concept of “stigma” is widely used in HIV/AIDS and other health-related fields, such as HIV-related stigma, drug-related stigma, and sexual-orientation-related stigma [47,48,49]. Therefore, we defined condom-related stigma as any taboos or misbeliefs about condom use or feeling ashamed or embarrassed to talk about using condoms, which was perceived by individuals at the individual, interpersonal, or social level [45]. Condom-related stigma contained the following four subthemes: (1) believing using a condom is a symbol of distrust, (2) believing using a condom is a symbol of HIV/STI prevention, (3) believing having a condom-related discussion is embarrassing, and (4) believing using a condom is a symbol of violating the traditional meaning of sexual intercourse [45]. However, there are no relevant scales to measure condom-related stigma.

After a literature review, we found that there were two scales related to condoms or condom use: the UCLA (University of California, Los Angeles) Multidimensional Condom Attitudes Scale (UCLA MCAS) [50] and the Condom Use Resistance Tactics Scale [51]. Although the UCLA MCAS also mentioned the stigmatizing attitude towards condoms or condom use (e.g., identity stigma, embarrassment about negotiating condom use and buying condoms), it was developed and used in heterosexual college students, and this scale focused on measuring their perception about the reliability and effectiveness of condoms (e.g., “Condoms are an effective method of birth control”) as well as the perceived sexual pleasure associated with condom use (e.g., “The use of condoms can make sex more stimulating”) [50]. The Condom Use Resistance Tactics Scale was developed and used in heterosexual men to explore what kinds of techniques they used to avoid using a condom with a woman who wanted to use one [51]; thus, it was not really an attitude-focused scale. While condom-related stigma is a type of negative attitude or symbolic attitude towards condoms and has more focus on MSM’s psychological perceptions of condoms, it cannot be measured by the above scales.

Therefore, based on the aforementioned operational definition (four subthemes), we developed a condom-related stigma scale and examined its psychometric properties among Chinese MSM. The condom-related stigma scale (CRSS) can be used to describe the prevalence and severity of condom-related stigma and evaluate the effectiveness of targeted behavioral interventions aiming to increase protected sex among MSM.

## 2. Materials and Methods

**Study Design**

The present study was conducted to develop an appropriate measure of condom-related stigma among MSM according to DeVellis’s scale development guidelines, which included the following steps: (1) determine clearly the concept to be measured, (2) generate an item pool, (3) determine the format for measurement, (4) have the initial item pool reviewed by experts, (5) administer items to a pilot sample, (6) consider the inclusion of validation items, (7) evaluate the items, and (8) optimize the scale length [52] (pp. 117–172). Since the **first step** was performed in our previous study [44,45], this study describes the following steps to develop the CRSS.

**Step 2: Generate an item pool**

We used the qualitative data from our previous meta-synthesis [44] to generate an item pool with 87 items. Then, our team brainstormed to reduce the item pool to 53 items.

**Step 3: Determine the format for measurement**

Based on the concept and operational definitions of condom-related stigma, the scale was developed as a self-reported instrument covering four domains: (1) perceived distrust; (2) perceived potential HIV/STI risk; (3) perceived embarrassment, and (4) perceived violation of the traditional understanding of sexual intercourse. The final dimensions of the scale were modified by the results of an exploratory factor analysis (EFA) and confirmed by a confirmatory factor analysis (CFA). The response to each item was rated on a 5-point Likert scale: “1 (strongly disagree), 2 (disagree), 3 (not sure), 4 (agree), and 5 (strongly agree)”; a higher score represented a higher level of condom-related stigma.

**Step 4: Have the initial item pool reviewed by experts**

We invited 5 experts, including one expert in the field of HIV/AIDS prevention and care, two psychologists experienced in instrument development, and two public health professionals, to assess the correctness, representativeness, readability, and appropriateness of the words used for each item. Two rounds of expert consultation were conducted. Based on each expert’s opinion, we reduced the number of items to 25 and rated the content validity of each question. Finally, in order to comprehensively capture the dimensions of the condom stigma and the richness of the item pool, we also performed a focus group interview with 30 participants who met the inclusion criteria to review the items, recommend any new reasonable items, and evaluate the readability, simplicity, and appropriateness of the items. The initial version of the CRSS was thus created (Table 1).

**Step 5: Administer items to a pilot sample**

**Participants**

Participants were eligible if the following applied: (1) they were male at birth; (2) they self-reported having sex with men in the previous year; (3) they were 18 years or older; and (4) they were able to read Chinese Standard Mandarin Language (for the 30 participants attending our focus group interview, they were able to read and speak Chinese Standard Mandarin Language). Participants were excluded if the following applied: (1) they self-reported a current mental disorder or (2) they were participating in other behavioral intervention studies.

**Measures**

Demographic information. Participants’ age, ethnicity, birthplace, monthly income, marital status, and education level were collected.

The CRSS (test version) consisted of 25 items with four domains, as described above.

Sexual behavior items. Participants were asked about their sexual practices in the past six months, including the frequency of condom use (e.g., *“How often have you used condoms while having sex in the last 6 months? [never/seldom/sometimes/often/always], Did you use condoms the last time you had anal/oral sex? [yes/no]”*) and HIV/AIDS and STI infection status (e.g., *“Have you ever been diagnosed with HIV/AIDS? [yes/no/never tested/no comments], Have you ever been diagnosed with any STI? [yes/no/never tested/no comments]”*).

HIV- and homosexuality-related stigma scales (Chinese version). This measure was originally developed by Bruce and then adapted for Chinese MSM by Liu [53]. It consists of 25 items with three domains: “public homosexual stigma” (10 items; e.g., “Many people unwillingly accept gay individuals”), “self-homosexual stigma” (8 items; e.g., “Sometimes I wish I were not gay”), and “public HIV stigma” (7 items; e.g., “HIV infected people should be ostracized by their spouse and family members”). Participants were asked to answer how much they agreed or disagreed with each item using a 4-point Likert scale. The highest possible scores for each subscale was 40, 32, and 28, respectively; the highest possible total score for the scale was 100. Higher scores indicated a higher level of perceived stigma. The Cronbach’s alpha coefficients were 0.85, 0.78, and 0.79 for public homosexual stigma, self-homosexual stigma, and public HIV stigma, respectively.

Sexual attitude scale (Chinese version). This measure was originally developed by Ou in China [54]. It consists of 18 items with four domains: “affirmativeness” (5 items; e.g., “Sex is a basic need for human beings”), “negativity” (5 items; e.g., “I feel embarrassed when I talk about sexual topics”), “openness” (4 items; e.g., “Casual sex is acceptable”), and “pro-homosexuality” (4 items; e.g., “Homosexual behavior is either morbid or abnormal”). Participants were asked to answer how much they agreed or disagreed with each item using a 5-point Likert scale. The highest possible scores for each subscale were 25, 25, 20, and 20, respectively; the highest possible total score for the scale was 90. Higher scores on each subscale indicated a higher level of affirmativeness/negativity/openness/pro-homosexuality on sexual attitudes, respectively. Some items were reverse-coded. The Cronbach’s alpha coefficients in the MSM population were 0.62, 0.73, 0.70, and 0.64 for affirmativeness, negativity, openness, and pro-homosexuality, respectively.

**Statistical analysis**

Statistical analyses were conducted using IBM SPSS Statistics version 26.0 and Amos version 24.0. SPSS was used to conduct descriptive statistics, item analysis, exploratory factor analysis, and psychometric properties testing, which included the Pearson correlation test and binary logistic regression analysis. Amos was used to perform CFA. The samples were split into two parts and stratified by randomization. The first half was utilized in item analysis, exploratory factor analysis, and psychometric properties testing (n = 216), and the second half was utilized in the CFA (n = 217).

**Step 6: Consider inclusion of validation items**

Item analysis was conducted to reduce the number of items. We deleted items that were non-significant on critical ratio (CR) or correlation between items and total scores. We also rejected items with correlation coefficients between items and total scores that were less than 0.40. We defined an acceptable loading strength of items on domains/factors to be ≥0.45 and an acceptable communality value to be ≥0.20. We decided which items should be retained by these criteria.

Exploratory factor analysis was conducted to determine factors by using IBM SPSS Statistics version 26.0 with principal axis factoring (PAF), Direct Oblimin rotation (as components were considered correlated), the Kaiser–Meyer–Olkin (KMO) test, the Bartlett’s test, a scree plot, and parallel analysis (PA). The professional book titled *Principles of exploratory factor analysis* indicated that “no single analysis is powerful enough to provide evidence of the viability of a factor structure”; thus, the final factors were determined by considering the above analyses, as well as the theoretical framework [55] (pp. 209–237).

**Step 7: Evaluate the items**

**Validity examination**

Content validity, convergent validity, empirical validity, and factorial validity were examined. Content validity was assessed by five experts as discussed above. An item-level content validity index (I-CVI), scale-level content validity index (S-CVI), random probability of chance agreement (*P_C_*), and modified kappa statistic (K*) were used to assess the content validity of the scale [56]. The calculation formulas were as follows: I-CVI = *A/n*; S-CVI = the mean of I-CVI of all items of the scale; $P_{C}=\frac{n!}{A!\left(n-A!\right)}\times 0.5^{n}$; $K^{*}=\frac{I-CVI-P_{C}}{1-P_{C}}$. Convergent validity was assessed using the Pearson correlation test in SPSS. The validated Chinese version of HIV- and homosexuality-related discrimination scales and the sexual attitude scale were used as the criterion to test the convergent validity, considering their relevance to condom-related stigma. Empirical validity was examined by using binary logistic regression analysis to assess the association between condom-related stigma and condomless sexual behavior. Factorial validity was assessed with EFA and CFA by using half of the data from sample testing for the SPSS analyses and half for the analyses conducted in Amos. For CFA, a maximum likelihood estimate (MLE) was used to fit the scale model. The ratio of chi-square degrees of freedom (χ^2^/df), the normalized fit index (NFI), the Tucker–Lewis index (TLI), the incremental fit index (IFI), the relative fit index (RFI), the comparative fit index (CFI), and the root mean square error of approximation (RMSEA) were used to evaluate the scale model. The χ^2^/df ratio should be less than 3 [57]; the NFI, TLI, IFI, RFI, and CFI should be greater than 0.90 [58]; and the RMSEA should be less than 0.08 [58] (pp. 212–260). The first-order model and the second-order model were revised repeatedly and verified again until they met the above index criteria.

**Reliability examination**

According to the Classical Test Theory [59], scale score reliability and test–retest reliability were used to examine the test error of this scale in terms of measurement content and measurement time. We performed scale reliability testing before and after item reduction in SPSS by examining the Cronbach’s alpha coefficient, split-half reliability, and correlation coefficient between items, factors, and the total scale. The Cronbach’s alpha coefficient should be above 0.70 for the purpose of developing a measurement tool [60] (pp. 159–265). The scale was separated into two halves by odd and even items to assess the split-half reliability. Test–retest reliability was assessed using the data from the 30 participants (randomly selected from all participants who meet the inclusion criteria) filling out the scale online at a two-week interval.

**Step 8: Optimize scale length**

The length of the scale was statistically derived through the whole process of scale development, including item reduction, validity, and reliability examination, since the good reliability and validity of the CRSS can prove that the scale length is optimized well [52]. In addition, we formed a committee (including the five experts mentioned earlier and our research team) to review and confirm the length of the scale.

**Ethical considerations**

This study was approved by the Ethics Committee of Xiangya Nursing School of Central South University, Changsha, Hunan (approval number: E201905). Participants were informed that involvement in the study was voluntary and anonymous. Electronic informed consent was obtained from each participant.

## 3. Results

### 3.1. Sample Characteristics

A total of 433 MSM were recruited. The average age was 25.6 years (SD = 0.319) with a range from 18 to 65 years old. Almost all participants (98.6%, n = 427) had a high school education or above. About half of the participants were single (50.8%, n = 220), born in rural areas (56.1%, n = 243), and had a monthly income less than RMB 5000 (USD 746) (57.3%, n = 248). More details can be found in Table 2.

### 3.2. Item Reduction

Twenty-five items were input into the item analysis. Three items (items 6, 11, and 19, see Table 3) were removed since their factor loadings were below 0.45. After removing these three items, the Cronbach’s alpha coefficient of the overall scale increased. Items 11 and 19 were also considered for deletion because their correlation coefficients with the total score of the scale were 0.300 and 0.399 (less than 0.40), respectively, with *p* < 0.05. The results of the item analysis can be found in Table 3.

**Figure 1:**
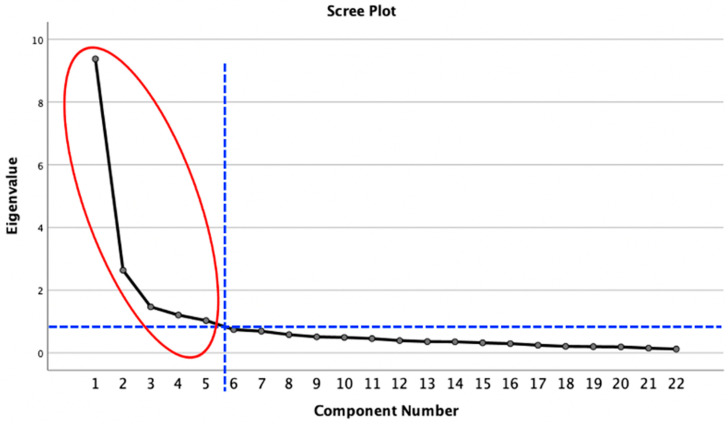
Scree plot of the common components.

In this study, we chose principal axis factoring (PAF), Direct Oblimin rotation, the Kaiser–Meyer–Olkin (KMO) test, the Bartlett’s test, a scree plot, and parallel analysis (PA) to conduct exploratory factor analysis on 22 items. The KMO was 0.906 (greater than 0.80 [60] (pp. 159–265)) and X^2^ was 3153.739 (*p* = 0.000 < 0.05) in Bartlett’s sphericity test, which indicated that there were common components among items and it was suitable for exploratory factor analysis. According to the result of the PAF and Direct Oblimin rotation (Table 4) (using the Kaiser–Guttman criterion: eigenvalue >1 [55] (pp. 209–237)) and the scree plot (Figure 1), five common components were retained. However, the results of the PA only indicated two common factors (Table 4), which could not be explained by our theoretical framework. In addition, Factor 5, containing items 14 and 15, was deleted after Direct Oblimin rotation since the items of a factor load should be equal to or greater than three, otherwise the content validity is not sufficient to measure the factor characteristics [60] (pp. 159–265). Combining the above analyses and our theoretical framework, we retained four common factors, containing twenty items.

The first factor containing items 1, 2, 3, 4, 5, 9, and 10 (items 1–7 in the final scale) was named “perceived distrust”; the second factor containing items 7, 8, 12, and 13 (items 8–11 in the final scale) was named “perceived potential HIV/STIs risk”; the third factor containing items 16, 17, 18, and 20 (items 12–15 in the final scale) was named “perceived embarrassment”; and the last factor containing items 21, 22, 23, 24, and 25 (items 16–20 in the final scale) was named “perceived violation of the traditional understanding of sexual intercourse” (Table 5).

### 3.3. Validity

#### 3.3.1. Content Validity

The I-CVI and K* of each item was equal to or greater than 0.80 and 0.76. The S-CVI of this scale was 0.99. Other results can be found in Table 6.

#### 3.3.2. Convergent Validity

The scores of items on four factors were significantly correlated with all comparative scales (Table 7).

#### 3.3.3. Empirical Validity

The empirical validity of the CRSS was greater than 0.70. Condom-related stigma predicted consistent condom use behavior during the previous six months (OR = 5.685, *p* < 0.05, 95% CI 3.618~8.935) and in the most recent instance of sexual intercourse (OR = 4.057, *p* < 0.05, 95%CI 2.281~7.216). The predictability of these behaviors in the regression model was 70.7% and 78.7%, respectively (Table 8 and Table 9).

#### 3.3.4. Factorial Validity

**Figure 2:**
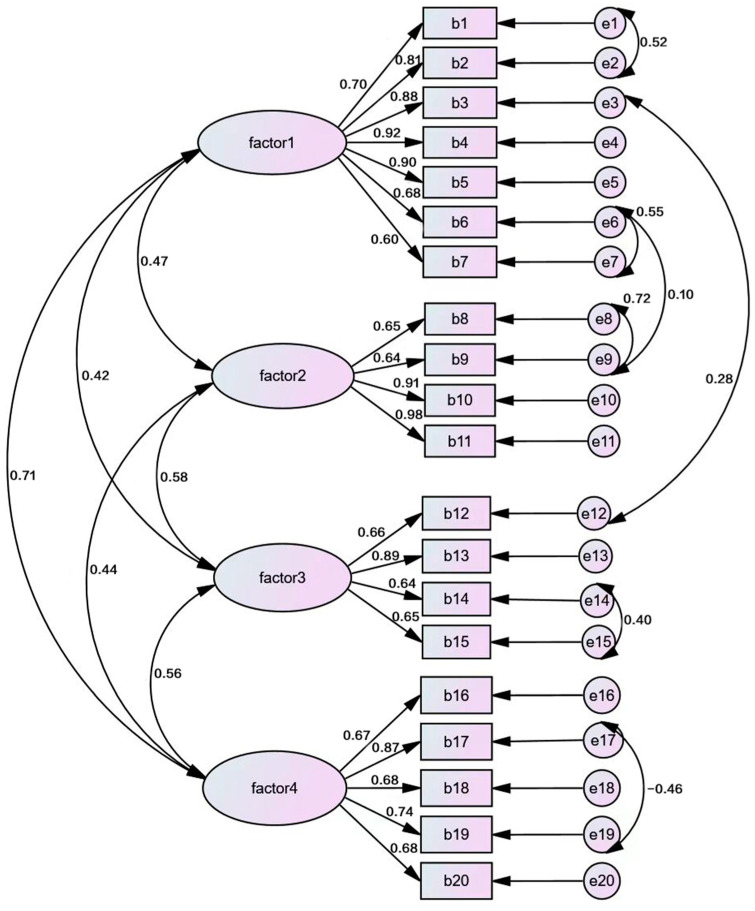
CFA first-order model of 20-item CRSS. Factor 1: perceived distrust; factor 2: perceived potential HIV/STI risk; factor 3: perceived embarrassment; factor 4: perceived violation of the traditional understanding of sexual intercourse.

**Figure 3:**
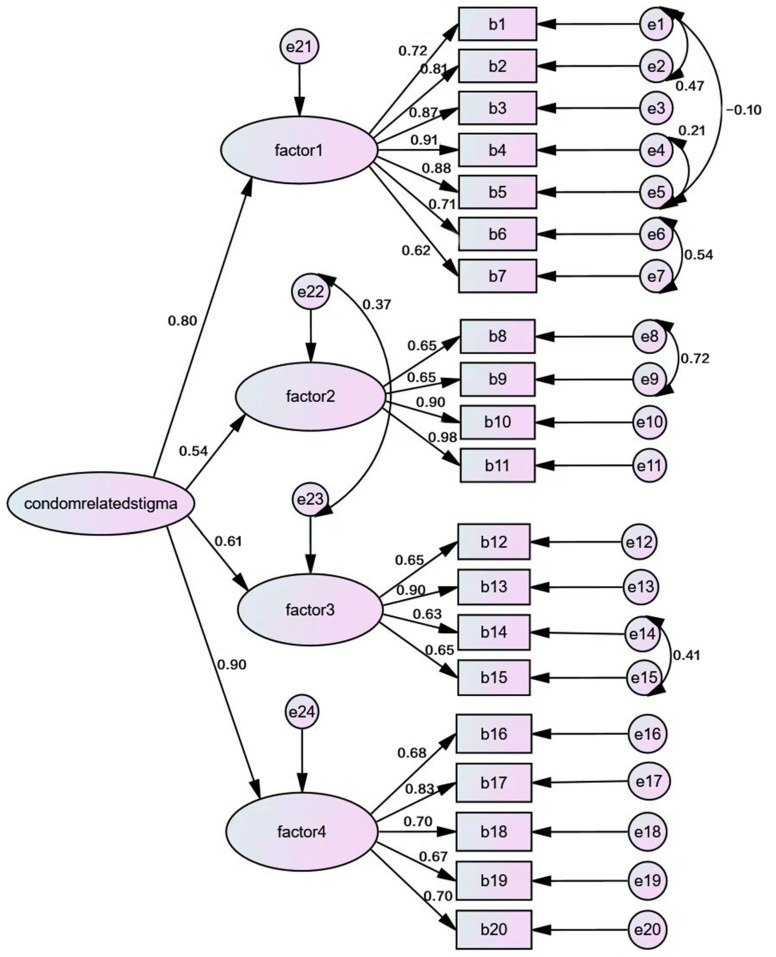
CFA second-order model of 20-item CRSS. Factor 1: perceived distrust; factor 2: perceived potential HIV/STI risk; factor 3: perceived embarrassment; factor 4: perceived violation of the traditional understanding of sexual intercourse.

Four factors were produced by exploratory factor analysis, which conformed to the original theoretical construct, explaining 68.624% of the variance. Using the four-factor model with 20 items, a CFA was conducted to cross-validate the fit of the data to the factor structure. Due to the correlation between the individual items (see Table 3) and the fact that some items tap into the same construct (based on our theoretical framework), we permitted the error terms to co-vary. Examination of modification indices indicated that co-varying the error terms for items 1 and 2; 3 and 12; 6 and 7; 6 and 9; 8 and 9; 14 and 15; and 17 and 19 improved the fit of the first-order model (Figure 2), and co-varying error terms for items 1 and 2; 1 and 5; 4 and 5; 6 and 7; 8 and 9; and 14 and 15 improved the fit of the second-order model (Figure 3). After co-varying the respective error terms, the indicators showed a relatively good fit to the model (χ2/df = 2.334, NFI = 0.887, TLI = 0.918, CFI = 0.932, RFI = 0.865, IFI = 0.932, RMSEA = 0.075) (Table 10).

### 3.4. Reliability

#### 3.4.1. Scale Score Reliability

The Cronbach’s alpha coefficient of the total scale was 0.926 and for each of the four subscales was 0.926, 0.877, 0.849, and 0.795, respectively. The split-half reliability of the total scale was 0.795 and for each of the four subscales was 0.899, 0.780, 0.844, and 0.750, respectively. The results of the exploratory factor analysis showed that, for the final 20-item scale, the correlation coefficients between each item and factor, between each factor, and between each factor and the total scale were all significant (all *p* < 0.05) (Table 3).

#### 3.4.2. Test–Retest Reliability

The test–retest reliability of the total scale was 0.950 (*p* < 0.05) and for the four subscales was 0.900, 0.909, 0.778, and 0.946 (all *p* < 0.05), respectively.

## 4. Discussion

This study presents the precise process of developing and psychometrically testing the CRSS, which aims to evaluate condom-related stigma perceived by Chinese MSM. It was culturally meaningful that the CRSS measured perceived distrust, perceived potential HIV/STI risk, perceived embarrassment, and perceived violation of the traditional understanding of sexual intercourse among Chinese MSM and that the scale reflected the operational definition of condom-related stigma. The scale can provide a sensitive indicator for evaluating strategies to reduce condom-related stigma and increase the practice of safer sex in Chinese cultural context, to contribute to HIV/AIDS prevention in China [61].

Factor 1 refers to the stigma caused by considering condom use or using condoms as a symbol of distrust and focuses on the individual’s attitude towards condom use, which was shaped by the subculture of the MSM community as well as the Chinese social–economic context. Affected by existing social norms, MSM prefer to protect their partner relationship with informal norms (e.g., social customs, personal morality, sexual mores, etc.) rather than formal norms (e.g., laws, regulations, etc.) [43]. Historically, condom use advocacy started for family planning in China and has been promoted along with HIV/AIDS prevention since the 1980s [62]. Therefore, condomless sex is used to illustrate the familiarity, intimacy, monogamy, commitment, and trust of a relationship in the traditional social understanding [38,40,63]. Items 1–7 reflect those beliefs, for example, “item 1: I would accept condomless sex if I trust my sexual partner”, “item 3: I think having condomless sex means having an intimate relationship with my sexual partner”, and “item 7: If I know my partner well enough, I would have condomless sex with him”.

Factor 2 refers to considering condom use as a symbol of HIV/STI prevention. Indeed, consistent condom use was officially promoted for the prevention and control of HIV/AIDS in the 1990s [64], for example in the 100% condom use project among key populations [18]. Thus, condoms are usually closely linked to HIV/AIDS prevention in people’s recognition. Moreover, HIV/AIDS-related stigma and moral-related stigma, such as having extra-relationship sex [4,36] are also attached to condom use, which are reflected in items 10–11 and 8–9, respectively.

Factor 3 refers to considering sex/condom-related discussion as an embarrassing topic. Affected by Confucianism, China is regarded as a comity and a courtesy nation, and people would feel shame talking about sex-related topics even in intimate relationships [65,66]. Therefore, sex/condom-related topics have always been considered taboo and uninformed in China [66]. Although attitudes towards sex have become more open since the “Reform and Open” policy [66], nowadays many people still feel embarrassed to talk about sex-related topics [67]. Items 12–15 reflect the above attitude, for example, “item 12: I am ashamed to talk about condom-related topics in my daily conversation” and “item 15: I would feel shy and shameful about carrying a condom”.

Factor 4 refers to the traditional perception about “sex” or “sexual behavior” when talking about condom use. In traditional Chinese culture, sexual behavior is regarded as the complete and unreserved passion of love [68] (pp. 26–30), and it is the direct combination of souls and bodies, which is reflected in items 16 and 17. Affected by the happiness philosophy of Taoism, people represented by Yang Zhu believe that human beings should break all etiquette rules and enjoy everything in the world; “sexual freedom” and “sexual indulgence” thus emerged [69], which is reflected in items 18 and 19. Confucianism believes that sex is the nature of human beings, and it is the most basic desire of people, which should be satisfied [65]. The desire of romantic and exciting sexual behavior is what people pursue and try their best to satisfy, which leads them to neglect condom use [68,70] (pp. 26–30). Item 20 illustrates this viewpoint.

Items analysis is a pivotal step in scale development [60] (pp. 159–265). Items in final scales should have high sensitivity, good representativeness, and strong scale score reliability [71] (pp. 485–492). In our study, qualitative and quantitative evaluation were used to analyze the items. The purpose of qualitative evaluation was to examine the content and expression of the items, while the quality of the items was examined by quantitative evaluation. The mixed methods evaluation supported the appropriateness of the items.

The results showed that the reliability of this scale was high. DeVellis [52] suggested that a scale with a Cronbach’s alpha coefficient between 0.70 and 0.80 was seen to be quite good and between 0.80 and 0.90 was seen to be very good. In this study, the Cronbach’s alpha coefficient of the scale was 0.926, and each subscale was between 0.795 and 0.926, which indicated that the scale had good reliability and all factors consistently measured the relevant construct. In general, it is better to have split-half reliability around or above 0.8 [60] (pp. 159–265). The split-half reliability of the total scale of this scale was 0.795, and the split-half reliability of each subscale was between 0.750 and 0.889, which indicated that the scale had good split-half reliability. In this study, retesting was performed at a two-week interval according to John’s recommendation [72] (pp. 16), and the results showed this scale had good stability across time.

The results of this study indicated the scale also has good validity. As for the content validity, Polit [73] suggested that K* ≥ 0.40–0.5 was fair, 0.60–0.74 was good, and greater than 0.74 was excellent; Davis [74] suggested that S-CVI ≥ 0.8 indicated that the content validity of the scale was good; Lynn [75] suggested that when the number of experts was less than or equal to 5, the I-CVI should be 1.00. Our results showed the good content validity of the scale. The CRSS was correlated with the two comparative scales, indicating good convergent validity. Additionally, the results of binary logistic regression analysis demonstrated that condom-related stigma was associated with condom use among MSM. This indicated that attitude towards condoms can affect condom use behavior, which is consistent with the conclusions of the study by Ramirez et al. [76]. Finally, both EFA and CFA were used to evaluate the factorial validity of the scale, and the results were satisfactory.

Several limitations should be noted. First, convenience sampling was used to recruit participants online, which may cause selection bias. Second, discriminant validity was not tested in our study and should be examined in future research. “Discriminant validity tests whether concepts or measurements that were not supposed to be related were actually unrelated” [77]. Average variance extracted (AVE) comparisons and the assessment of the HTMT (heterotrait–monotrait ratio) (the cutoff of the HTMT was 0.85) were recommended approaches to test for discriminant validity on the construct level [78,79], while exploratory factor analysis was a recommended approach to test for discriminant validity on the item level [80]. Finally, this scale was a culture-adaptive scale rooted in the Chinese cultural context, and it will need to be adapted if it will be used in other country or cultural contexts.

## 5. Conclusions

A 20-item self-reported CRSS with four factors (perceived distrust, perceived potential HIV/STI risk, perceived embarrassment, and perceived violation of the traditional understanding of sexual intercourse) has been developed and is a valid, reliable, and culture-adaptive scale to measure condom-related stigma in China. Future interventions to reduce condomless sex can not only target HIV/AIDS-related knowledge and beliefs but, more importantly, could target condom-related stigma. This study adds a new tool for safer-sex interventions to prevent HIV/AIDS among the Chinese MSM population.

## Figures and Tables

**Table 1:** The initial version of the CRSS.

Item		Strongly Disagree 非常不同意	Disagree 不同意	Not Sure 不确定	Agree 同意	Strongly Agree 非常同意
**Domain 1: perceived distrust 信任关系符号化**						
1.	I would accept condomless sex if I trust my sexual partner. 基于对性伴的信任，我会接受无套性行为。	1	2	3	4	5
2.	Based on the trust in my sexual partner, I would take the initiative to ask for condomless sex. 基于对性伴的信任，我会主动提出无套要求。	1	2	3	4	5
3.	I think having condomless sex means having an intimate relationship with my sexual partner. 我认为无套意味着我和性伴关系亲密。	1	2	3	4	5
4.	I would express my love to my sexual partner through having sex without a condom. 我会通过无套来表达我对性伴的爱。	1	2	3	4	5
5.	I would express our monogamous relationship to my boyfriend/girlfriend/wife through having sex without a condom. 我会通过无套来表达我对男朋友/女朋友/妻子的专一。	1	2	3	4	5
6.	If my sexual partner carries a condom with him, I would suspect that he has multiple sexual partners. 如果性伴随身携带安全套，我会怀疑他近期内有多个性伴。	1	2	3	4	5
7.	If my boyfriend/girlfriend/wife suggests condom use, I would suspect that he/she has sex with other people. 如果我的男朋友/女朋友/妻子提出使用安全套，我会怀疑他/她还和其他人发生性关系。	1	2	3	4	5
8.	If I suggest condom use to my boyfriend/girlfriend/wife, I would be suspected of having sex with someone else. 如果我向男朋友/女朋友/妻子提出使用安全套，我会被怀疑还和其他人发生性关系。	1	2	3	4	5
9.	If my sexual partner gives me a commitment to our relationship, I would have condomless sex with him. 如果性伴给予我确定情侣关系的承诺，我会与他发生无套性行为。	1	2	3	4	5
10.	If I know my sexual partner well enough, I would have condomless sex with him. 如果我足够了解、熟悉我的性伴，我会与他发生无套性行为。	1	2	3	4	5
**Domain 2: perceived potential HIV/STIs risk 艾滋病/性病符号化**						
11.	I think using condoms is purely for the prevention of HIV/AIDS and STIs. 我认为使用安全套纯粹是为了预防艾滋病和性病。	1	2	3	4	5
12.	If my sexual partner asks to use a condom, I would suspect that he is infected with HIV/AIDS or other STIs. 如果性伴提出使用安全套，我会怀疑他感染了艾滋病或其它性病。	1	2	3	4	5
13.	If I ask to use a condom, I would be suspected of infection of HIV/AIDS or other STIs. 如果我提出使用安全套，我会被怀疑感染了艾滋病或其它性病。	1	2	3	4	5
14.	Because HIV/AIDS can be controlled by medication, I am willing to bear the risk of contracting HIV/AIDS due to condomless sex. 因为艾滋病可以通过药物控制，所以我愿意承担因无套而感染艾滋病的风险。	1	2	3	4	5
15.	I think during sex, the “top” sexual role (inserter) does not need to use condoms. 我认为性行为中，插入方可以不使用安全套。	1	2	3	4	5
**Domain 3: perceived embarrassment 性话题的羞耻化**						
16.	I am ashamed to talk about condom-related topics in my daily conversation. 日常交流中，我羞于讨论安全套的话题。	1	2	3	4	5
17.	I would acquiesce to having condomless sex due to being ashamed to talk about sex or condom-related topics. 我会因羞于谈论性或安全套相关的话题而默认发生无套行为。	1	2	3	4	5
18.	I think it’s embarrassing to try to figure out some condom-related knowledge, such as “what kind of condoms to buy” or “how to use condoms”. 我认为尝试去弄清楚安全套的相关知识（如应该购买哪种、如何使用）是一件很尴尬的事。	1	2	3	4	5
19.	I am afraid of the embarrassment caused by the discovery of my condoms by my parents or others. 我害怕自己的安全套被父母或其他人发现而引发尴尬。	1	2	3	4	5
20.	I would feel shy and shameful about carrying a condom. 我会因为携带安全套而感到害羞。	1	2	3	4	5
**Domain 4: perceived violation of the traditional understanding of sexual intercourse 传统性认知符号化**						
21.	I think “real sex” requires a direct genital-anal contact. 我认为“真正的性行为”是生殖器-肛门之间的直接接触。	1	2	3	4	5
22.	I think ejaculating semen into the sexual partner’s body is a necessary part of having sex. 我觉得将精液射入性伴体内是性行为必须有的环节。	1	2	3	4	5
23.	I think sex should be free and unfettered (by using condoms). 我认为性行为应该是自由的，不被任何东西束缚的。	1	2	3	4	5
24.	I don’t think the sexual process should be interrupted because of wearing condoms. 我认为性行为过程不应该因为戴安全套而被中断。	1	2	3	4	5
25.	I think sex should be romantic and exciting, and I’m willing to risk it (having condomless sex). 我认为性行为应该是浪漫的、刺激的，我甘愿为之冒险。	1	2	3	4	5

Sexual partner: refers to the people who you have sex with, including boyfriends, girlfriends, wives, casual partners, commercial partners, etc. Boyfriend/girlfriend/wife: refers to the people with whom you have a committed relationship.

**Table 2:** Participant characteristics (N = 433).

	Group	*N*	Proportion (*%*)
Age (year)	≤29	339	78.3
	30~39	74	17.1
	40~49	15	3.5
	≥50	5	1.1
Ethnicity	Han	400	92.4
	Minority	33	7.6
Birthplace	countryside	243	56.1
	City	190	43.9
Monthly income (RMB: yuan)	<2000	104	24.0
	2000~5000	144	33.3
	5000~10,000	139	32.1
	>10,000	46	10.6
Marital status	Not a having female (male) partner	220	50.8
	Having a female (male) partner but not cohabiting with	136	31.4
	Cohabiting with a female (male) partner	58	13.4
	Married	12	2.8
	Divorced	5	1.2
	Widowed	2	0.5
Education	Junior school or lower	6	1.4
	High school	39	9.0
	Training school	107	24.7
	Undergraduate	235	54.3
	Graduate or above	46	10.6

**Table 3:** Summary of item analysis for the test-version CRSS.

Item	Extreme Group Comparison	Correlation between Individual Items Score and the Total Score		Homogeneity Test			Number of Substandard Indicators	Notes
	CR	Initial Items	Corrected Items	Cronbach’s α after Item Deletion	Communality	Factor Loading		
b1	12.795 ***	0.643 ***	0.595	0.922	0.406	0.638	0	Retained
b2	14.215 ***	0.691 ***	0.652	0.921	0.478	0.692	0	Retained
b3	14.683 ***	0.699 ***	0.656	0.921	0.484	0.696	0	Retained
b4	14.801 ***	0.776 ***	0.747	0.919	0.621	0.788	0	Retained
b5	13.036 ***	0.702 ***	0.665	0.921	0.502	0.708	0	Retained
b6	5.583 ***	0.436 ***	^#^ 0.376	^#^ 0.926	^#^ 0.168	^#^ 0.409	4	Deleted
b7	10.106 ***	0.636 ***	0.595	0.922	0.413	0.643	0	Retained
b8	9.212 ***	0.625 ***	0.586	0.922	0.404	0.636	0	Retained
b9	14.042 ***	0.736 ***	0.703	0.920	0.543	0.737	0	Retained
b10	13.530 ***	0.633 ***	0.584	0.922	0.376	0.613	0	Retained
b11	3.969 ***	^#^ 0.300 ***	^#^ 0.220	^#^ 0.929	^#^ 0.055	^#^ 0.234	5	Deleted
b12	8.110 ***	0.629 ***	0.590	0.922	0.438	0.661	0	Retained
b13	9.405 ***	0.709 ***	0.678	0.921	0.542	0.736	0	Retained
b14	7.092 ***	0.596 ***	0.559	0.923	0.407	0.638	0	Retained
b15	9.373 ***	0.639 ***	0.608	0.922	0.455	0.675	0	Retained
b16	7.897 ***	0.527 ***	0.481	0.924	0.273	0.523	3	Retained
b17	10.856 ***	0.694 ***	0.665	0.921	0.500	0.707	0	Retained
b18	7.624 ***	0.497 ***	0.447	0.924	0.240	0.490	0	Retained
b19	5.371 ***	^#^ 0.399 ***	^#^ 0.333	^#^ 0.927	^#^ 0.119	^#^ 0.345	5	Deleted
b20	7.387 ***	0.573 ***	0.526	0.923	0.301	0.549	0	Retained
b21	9.352 ***	0.574 ***	0.519	0.923	0.298	0.546	0	Retained
b22	12.107 ***	0.725 ***	0.695	0.921	0.548	0.740	0	Retained
b23	9.088 ***	0.549 ***	0.496	0.923	0.289	0.538	0	Retained
b24	11.776 ***	0.611 ***	0.566	0.922	0.374	0.612	0	Retained
b25	10.548 ***	0.640 ***	0.606	0.922	0.447	0.669	0	Retained
**Criteria**	**≥3.000**	**≥0.400**	**≥0.400**	**≤0.925**	**≥0.200**	**≥0.450**		

0.925 is the Cronbach’s α of the initial condom stigma scale; ^#^ means not reaching the criteria; *** means *p* < 0.001 (two-tailed); n.s. means not significant.

**Table 4:** The results of the principal axis factoring, Direct Oblimin rotation, and parallel analysis.

Component	Initial Eigenvalues			Extraction Sums of Squared Loadings			Parallel Analysis
	Total	% of Variance	Cumulative %	Total	% of Variance	Cumulative %	Percentile Eigenvalue (95th)
1	9.376	42.616	42.616	9.376	42.616	42.616	1.724
2	2.634	11.974	54.590	2.634	11.974	54.590	1.586
3	1.466	6.664	61.254	1.466	6.664	61.254	1.492
4	1.205	5.478	66.732	1.205	5.478	66.732	1.416
5	1.029	4.679	71.411	1.029	4.679	71.411	1.349
6	0.741	3.370	74.781				1.284
……	……	……	……				……
22	0.121	0.550	100.000				0.556

Extraction method: principal axis factoring. When factors are correlated, sums of squared loadings cannot be added to obtain a total variance.

**Table 5:** The final version of the CRSS.

Item		Strongly Disagree 非常不同意	Disagree 不同意	Not Sure 不确定	Agree 同意	Strongly Agree 非常同意
**Domain 1: perceived distrust 信任关系符号化**						
1.	I would accept condomless sex if I trust my sexual partner. 基于对性伴的信任，我会接受无套性行为。	1	2	3	4	5
2.	Based on the trust in my sexual partner, I would take the initiative to ask for condomless sex. 基于对性伴的信任，我会主动提出无套要求。	1	2	3	4	5
3.	I think having condomless sex means having an intimate relationship with my sexual partner. 我认为无套意味着我和性伴关系亲密。	1	2	3	4	5
4.	I would express my love to my sexual partner through having sex without a condom. 我会通过无套来表达我对性伴的爱。	1	2	3	4	5
5.	I would express our monogamous relationship to my boyfriend/girlfriend/wife through having sex without a condom. 我会通过无套来表达我对男朋友/女朋友/妻子的专一。	1	2	3	4	5
6.	If my sexual partner gives me a commitment to our relationship, I would have condomless sex with him. 如果性伴给予我确定情侣关系的承诺，我会与他发生无套性行为。	1	2	3	4	5
7.	If I know my partner well enough, I would have condomless sex with him. 如果我足够了解、熟悉我的性伴，我会与他发生无套性行为。	1	2	3	4	5
**Domain 2: perceived potential HIV/STIs risk 艾滋病/ 性病符号化**						
8.	If my boyfriend/girlfriend/wife suggests condom use, I would suspect that he/she has sex with other people. 如果我的男朋友/女朋友/妻子提出使用安全套，我会怀疑他还和其他人发生性关系。	1	2	3	4	5
9.	If I suggest condom use to my boyfriend/girlfriend/wife, I would be suspected of having sex with someone else. 如果我向男朋友/女朋友/妻子提出使用安全套，我会被怀疑还和其他人发生性关系。	1	2	3	4	5
10.	If my sexual partner asks to use a condom, I would suspect that he is infected with HIV/AIDS or other STIs. 如果性伴提出使用安全套，我会怀疑他感染了艾滋病或其它性病。	1	2	3	4	5
11.	If I ask to use a condom, I would be suspected of infection of HIV/AIDS or other STIs. 如果我提出使用安全套，我会被怀疑感染了艾滋病或其它性病。	1	2	3	4	5
**Domain 3: perceived embarrassment 性话题的羞耻化**						
12.	I am ashamed to talk about condom-related topics in my daily conversation. 日常交流中，我羞于讨论安全套的话题。	1	2	3	4	5
13.	I would acquiesce to having condomless sex due to being ashamed to talk about sex or condom-related topics. 我会因羞于谈论性或安全套相关的话题而默认发生无套行为。	1	2	3	4	5
14.	I think it’s embarrassing to try to figure out some condom-related knowledge, such as “what kind of condoms to buy” or “how to use condoms”. 我认为尝试去弄清楚安全套的相关知识（如应该购买哪种、如何使用）是一件很尴尬的事。	1	2	3	4	5
15.	I would feel shy and shameful about carrying a condom. 我会因为携带安全套而感到害羞。	1	2	3	4	5
**Domain 4: perceived violation of the traditional understanding of sexual intercourse 传统性认知符号化**						
16.	I think “real sex” requires a direct genital-anal contact. 我认为“真正的性行为”是生殖器-肛门之间的直接接触。	1	2	3	4	5
17.	I think ejaculating semen into the sexual partner’s body is a necessary part of having sex. 我觉得将精液射入性伴体内是性行为必须有的环节。	1	2	3	4	5
18.	I think sex should be free and unfettered (by using condoms). 我认为性行为应该是自由的，不被任何东西束缚的。	1	2	3	4	5
19.	I don’t think the sexual process should be interrupted because of wearing condoms. 我认为性行为过程不应该因为戴安全套而被中断。	1	2	3	4	5
20.	I think sex should be romantic and exciting, and I’m willing to risk it (having condomless sex). 我认为性行为应该是浪漫的、刺激的，我甘愿为之冒险。	1	2	3	4	5

Sexual partner: it refers to the people who you have sex with, including boyfriends, girlfriends, wives, casual partners, commercial partners, etc. Boyfriend/girlfriend/wife: it refers to the people with whom you have a committed relationship.

**Table 6:** Calculation of expert score and content validity index for the 20-item CRSS.

Items	Expert Score					Number of Experts Scoring 3 or 4	I-CVI	*Pc*	K*	Evaluation
	A	B	C	D	F					
1	4	4	4	4	4	5	1	0.041	1	Excellent
2	4	4	4	4	4	5	1	0.041	1	Excellent
3	4	4	4	4	4	5	1	0.041	1	Excellent
4	4	4	4	4	4	5	1	0.041	1	Excellent
5	4	4	4	4	4	5	1	0.041	1	Excellent
6	4	3	2	4	3	4	0.8	0.156	0.76	Excellent
7	4	4	4	4	4	5	1	0.041	1	Excellent
8	4	4	4	4	4	5	1	0.041	1	Excellent
9	4	4	4	4	3	5	1	0.041	1	Excellent
10	4	4	4	4	4	5	1	0.041	1	Excellent
11	4	4	4	4	4	5	1	0.041	1	Excellent
12	4	4	4	4	3	5	1	0.041	1	Excellent
13	4	4	4	4	4	5	1	0.041	1	Excellent
14	4	4	4	4	4	5	1	0.041	1	Excellent
15	4	4	4	4	4	5	1	0.041	1	Excellent
16	4	4	4	4	4	5	1	0.041	1	Excellent
17	4	4	4	4	4	5	1	0.041	1	Excellent
18	4	4	4	4	3	5	1	0.041	1	Excellent
19	4	4	4	4	3	5	1	0.041	1	Excellent
20	4	4	4	4	4	5	1	0.041	1	Excellent

I-CVI = *A*/*n*; S-CVI = the mean of I-CVI of all items of the scale; $P_{C}=\frac{n!}{A!\left(n-A!\right)}\times 0.5^{n}$; $K^{*}=\frac{I-CVI-P_{C}}{1-P_{C}}$.

**Table 7:** Correlation of domains/subscales with HIV- and homosexuality-related stigma and sexual attitudes (Pearson correlation coefficient).

		Factor 1	Factor 2	Factor 3	Factor 4
HIV- and homosexuality-related stigma	Public homosexual stigma	0.175 *	0.074	0.148 *	0.161 *
	Self-homosexual stigma	0.242 **	0.310 **	0.457 **	0.237 **
	AIDS-related discrimination	0.272 **	0.276 **	0.326 **	0.343 **
	Total score	0.292 **	0.272 **	0.395 **	0.307 **
Sexual attitudes	Affirmativeness	0.122	−0.142 **	−0.151 **	−0.016
	Negativity	0.223 **	0.349 **	0.516 **	0.295 **
	Openness	0.284 **	0.176 **	0.184 **	0.288 **
	Pro-homosexuality	−0.067	−0.286 **	−0.303	−0.238 **
	Total score	0.329 **	0.100 **	0.197 **	0.218 **

**: At the 0.01 level (two-tailed), the correlation was statistically significant. *: At the 0.05 level (two-tailed), the correlation was statistically significant.

**Table 8:** Predicted results of condom use in the past 6 months’ sexual behaviors (Y_1_) in MSM by assessing condom-related stigma.

			Prediction Y_1_^1^		%
			Consistent Condom Use	Inconsistent Condom Use	
Reality Y_1_		Consistent condom use	165	70	70.2
		Inconsistent condom use	51	127	71.3
%					70.7

^1^ Y_1_: Condom use in the past 6 months’ sexual behaviors.

**Table 9:** Predicted results of condom use in the recent sexual behavior (Y_2_) in MSM by assessing condom-related stigma.

		Prediction Y_2_^1^		%
		Consistent Condom Use	Inconsistent Condom Use	
Reality Y_2_	Consistent condom use	325	3	99.1
	Inconsistent condom use	85	0	0
%				78.7

^1^ Y_2_: Condom use in the recent sexual behavior.

**Table 10:** First-order model and second-order model fit of confirmatory factor analysis in MSM group.

Indicators	Standards	Test Value in the First-Order Model		Test Value in the Second-Order Model	
		Before Modification	After Modification	Before Modification	After Modification
χ2/df	<3	4.065	2.145	4.206	2.334
NFI	>0.90	0.797	0.898	0.788	0.887
TLI	>0.90	0.812	0.930	0.804	0.918
CFI	>0.90	0.838	0.942	0.828	0.932
RFI	>0.90	0.765	0.876	0.757	0.865
IFI	>0.90	0.839	0.943	0.830	0.932
RMSEA	<0.08	0.119	0.073	0.122	0.075

## Data Availability

Not applicable.
